# Robust solutions to box-constrained stochastic linear variational inequality problem

**DOI:** 10.1186/s13660-017-1529-2

**Published:** 2017-10-10

**Authors:** Mei-Ju Luo, Yan Zhang

**Affiliations:** School of Mathematics, Liaoning University, Liaoning, 110036 China

**Keywords:** 90C33, 90C30, box-constrained, stochastic variational inequality, linear, robust solution

## Abstract

We present a new method for solving the box-constrained stochastic linear variational inequality problem with three special types of uncertainty sets. Most previous methods, such as the expected value and expected residual minimization, need the probability distribution information of the stochastic variables. In contrast, we give the robust reformulation and reformulate the problem as a quadratically constrained quadratic program or convex program with a conic quadratic inequality quadratic program, which is tractable in optimization theory.

## Introduction

Variational inequality theory is an important branch in operations research. Recall that the variational inequality problem, denoted by $\operatorname{VI}(X,F)$, is to find a vector $x^{\ast}\in X$ such that

1 $$\begin{aligned} \bigl(x-x^{\ast} \bigr)^{T}F \bigl(x^{\ast} \bigr)\geq0, \quad \forall x\in X, \end{aligned}$$

where $X\subseteq R^{n}$ is a non-empty closed convex set, $F:X \rightarrow R^{n}$ is a given function. A particularly important class of $\operatorname{VI}(X,F)$ is the box-constrained variational inequality problem (see [1]), denoted by $\operatorname{VI}(l,u,F)$, where $X=D=\{x\in R^{n} \mid l\leq x\leq u\}$, $l:=(l_{1},\ldots,l_{n})^{T}, u:=(u_{1},\ldots,u_{n})^{T}$, along with the lower bounds $l_{i}\in R \cup\{-\infty\}$, the upper bounds $u_{i}\in R \cup\{+\infty\}$ and $l_{i}< u_{i}$ for all $i=1,\ldots,n$. To obtain the solutions of VI($l,u,F$), many methods are presented based on the KKT system, which is given as follows:

2 $$\begin{aligned} \begin{aligned} &x_{i}-l_{i}\geq0,\qquad F_{i}(x)+y_{i} \geq 0,\qquad (x_{i}-l_{i}) \bigl(F_{i}(x)+y_{i} \bigr)=0, \\ &u_{i}-x_{i}\geq0, \qquad y_{i}\geq0,\qquad (u_{i}-x_{i})y_{i}=0. \end{aligned} \end{aligned}$$

Note that this KKT system is a complementarity problem in fact.

The box-constrained variational inequality problem has many applications ranging from operations research to economic equilibrium and engineering problems [2]. However, some elements may involve uncertain data in practice. For example, the demands are generally difficult to be determined in supply chain network, because they vary with the change of income level [3, 4]. Moreover, in traffic equilibrium problems, the selfish users’ attempt to minimize travel cost leads the equilibrium (or steady-state) flows to uncertainty [5–8].

For the existence of stochastic elements, the solutions of stochastic variational inequality problem would change with stochastic elements respectively. In order to meet the needs in practice, many researchers begin to consider the following stochastic variational inequality problem, denoted by $\operatorname{SVI}(X,F)$, which requires an $x^{\ast}$ such that

3 $$\begin{aligned} \bigl(x-x^{\ast} \bigr)^{T}F \bigl(x^{\ast}, \omega \bigr)\geq0, \quad\forall x\in X, \omega\in \Omega, \mbox{ a.s.}, \end{aligned}$$

where $\omega\in\Omega\subseteq R^{\tau}$ is a stochastic vector and $a.s$. is the abbreviation for ‘almost surely’ under the given probability measure. Particularly, if $F(x,\omega):=M(\omega)x+q(\omega )$ and $X=D=\{x\in R^{n},l\leq x\leq u\}$ is a non-empty set, where $M(\omega):\Omega\rightarrow R^{n\times n}$ and $q(\omega):\Omega \rightarrow R^{n}$ are mappings with respect to *ω*, we then call problem (3) box-constrained stochastic linear variational inequality problem, denoted by $\operatorname{SLVI}(l,u,F)$. Following from (2), the KKT system of $\operatorname{SLVI}(l,u,F)$ can be rewritten as

4 $$\begin{aligned} 0\leq \begin{pmatrix} t \\ y \end{pmatrix}\perp \begin{bmatrix} M(\omega) & I \\ -I & \mathbf{0} \end{bmatrix} \begin{pmatrix} t \\ y \end{pmatrix} +\begin{pmatrix} M(\omega)l+q(\omega) \\ u-l \end{pmatrix}\geq0, \end{aligned}$$

where $t=x-l\in R^{n}_{+}$ and $\bigl({\scriptsize\begin{matrix}{} t \cr y \end{matrix}} \bigr) \in R^{2n}_{+}$ are vectors, *I* denotes an *n*-dimensional identify matrix and **0** represents a zero matrix with suitable dimension.

Before proceeding, we briefly touch upon earlier efforts about SVI($X,F$). For example, the expected value (EV) method [9–11] and the expected residual minimization (ERM) method [12–14] focused on minimizing the average or the average of expected residual. These methods needed the information of a probability distribution and focused on providing estimators of local solutions. In the spirit of robust approaches, instead of ERM or EV, we consider the minimization of the worst-case residual over a particular uncertainty set Ω.

By employing KKT system (4), we give the robust reformulation of $\operatorname{SLVI}(l,u,F)$ as follows:

5 $$\begin{aligned} \begin{aligned} &\min \mathop{\max} _{\omega\in\Omega} \bigl(t^{T},y^{T} \bigr) \left( \begin{bmatrix} M(\omega) & I \\ -I & \mathbf{0} \end{bmatrix} \begin{pmatrix} t \\ y \end{pmatrix} + \begin{pmatrix} M(\omega)l+q(\omega) \\ u-l \end{pmatrix} \right), \\ &\quad \mbox{s.t.}\quad \mathop{\min} _{\omega\in\Omega} \begin{bmatrix} M(\omega) & I \\ -I & \mathbf{0} \end{bmatrix} \begin{pmatrix} t \\ y \end{pmatrix} + \begin{pmatrix} M(\omega)l+q(\omega) \\ u-l \end{pmatrix} \geq0, \\ &\quad\quad\quad\quad t,y\geq0. \end{aligned} \end{aligned}$$

Obviously, (5) can be rewritten as follows:

6 $$\begin{aligned} \begin{aligned} &\min \quad\quad\quad z \\ & \mbox{s.t.} \quad\mathop{\max} _{\omega\in\Omega} \bigl(t^{T},y^{T} \bigr) \left( \begin{bmatrix} M(\omega) & I \\ -I & \mathbf{0} \end{bmatrix} \begin{pmatrix} t \\ y \end{pmatrix} + \begin{pmatrix} M(\omega)l+q(\omega) \\ u-l \end{pmatrix} \right)\leq z, \\ & \quad\quad\mathop{\min} _{\omega\in\Omega} \begin{bmatrix} M(\omega) & I \\ -I & \mathbf{0} \end{bmatrix} _{j} \begin{pmatrix} t \\ y \end{pmatrix} +\begin{pmatrix} M(\omega)l+q(\omega) \\ u-l \end{pmatrix} _{j} \geq0,\quad \forall j=1, \ldots, 2n, \\ &\quad\quad\quad t,y\geq0. \end{aligned} \end{aligned}$$

Note that $\bigl( {\scriptsize\begin{matrix}{} t \cr y \end{matrix}} \bigr)\geq0 $ solves (4) if and only if $\bigl({\scriptsize\begin{matrix}{} t \cr y\end{matrix}} \bigr)$ is a solution of (5) with optimal value zero by Lemma 3.1 in [15].

The organization of this paper is as follows. In Section 2, we discuss the tractable robust counterparts of monotone $\operatorname{SLVI}(l,u,F)$ in different uncertain sets. Non-monotone generalizations and their tractable robust counterparts form the core of Section 3. In Section 4, we give the conclusions.

## Tractable robust counterparts of monotone $\operatorname{SLVI}(l,u,F)$

To begin with, we provide robust counterparts in regimes where $M(\omega ^{1})$ is a stochastic positive semidefinite matrix and $q(\omega^{2})$ is a stochastic vector with $\omega^{1}\in\Omega^{1}$ and $\omega^{2}\in \Omega^{2}$, where $\Omega^{1}$ and $\Omega^{2}$ are uncertainty sets. $\Omega^{1}$ contains $\Omega^{1}_{\infty}$ and $\Omega^{1}_{1}$. These two types of uncertainty sets state as follows:

$$\begin{aligned} \Omega^{1}_{\infty}= \bigl\{ \omega: \Vert \omega \Vert _{\infty}\leq1,\omega\geq0 \bigr\} \quad \mbox{and} \quad \Omega^{1}_{1}= \bigl\{ \omega: \Vert \omega \Vert _{1}\leq1,\omega\geq0 \bigr\} . \end{aligned}$$

$\Omega^{2}$ contains $\Omega^{2}_{\infty}$, $\Omega^{2}_{1}$ and $\Omega^{2}_{2}$, these three types of uncertainty sets state as follows:

$$\begin{aligned} \Omega^{2}_{\infty}= \bigl\{ \omega: \Vert \omega \Vert _{\infty}\leq1 \bigr\} ,\qquad \Omega ^{2}_{1}= \bigl\{ \omega: \Vert \omega \Vert _{1}\leq1 \bigr\} \quad\mbox{and}\quad \Omega^{2}_{2}= \bigl\{ \omega: \Vert \omega \Vert _{2}\leq1 \bigr\} . \end{aligned}$$

Here, $\Vert \cdot \Vert _{\infty}, \Vert \cdot \Vert _{1}, \Vert \cdot \Vert _{2}$ denotes infinite norm, 1-norm and 2-norm, respectively. Moreover, we define $M(\omega^{1})=M_{0}+\sum_{s = 1}^{S} {\omega_{s}^{1}M_{s} }, q(\omega ^{2})=q_{0}+\sum_{s = 1}^{S} {\omega_{s}^{2} q_{s}}$, where $M_{s}, s=1,\ldots,S$, is an *n*-dimensional symmetric positive semidefinite matrix and $q_{s}, s=1,\ldots,S$, is an *n*-dimensional vector. Without loss of generality, we assume that $M(\omega^{1})$ is symmetric; if not, we may replace the matrixes by their symmetrized counterparts. This assumption guarantees $\bigl[ {\scriptsize\begin{matrix}{} M(\omega) & I \cr -I & \mathbf{0} \end{matrix}} \bigr]\bigl( {\scriptsize\begin{matrix}{} t \cr y \end{matrix}} \bigr)+\bigl( {\scriptsize\begin{matrix}{} M(\omega)l+q(\omega) \cr u-l\end{matrix}} \bigr)$ is monotone on $R^{2n}_{+}$ (see [16]).

In this section, we first consider the case when $\Omega^{1}=\Omega _{\infty}^{1}$, the robust counterpart of $\operatorname{SLVI}(l,u,F)$, and then focus on the case $\Omega^{1}=\Omega^{1}_{1}$ while $\omega^{2}\in\Omega ^{2}$. We now prove that robust problem (6) can be reformulated as a quadratically constrained quadratic program (QCQP) or convex program with a conic quadratic inequality quadratic program [17, 18] under the different uncertain sets.

### Theorem 1

*Consider the optimization problem* (6) *with* $\omega^{1}\in\Omega _{\infty}^{1}$ *and* $M(\omega^{1})=M_{0}+\sum_{s = 1}^{S} {\omega _{s}^{1}M_{s} }, q(\omega^{2})=q_{0}+\sum_{s = 1}^{S} {\omega_{s}^{2} q_{s}}$. *Then* (6) *can be reformulated respectively as QCQP or convex program with a conic quadratic inequality quadratic program while* $\omega^{2}$ *belongs to different sets of* $\Omega^{2}$.

### Proof

Case 1: $\Omega^{2}=\Omega^{2}_{\infty}$.

We first give the equivalent reformulation of the first constraint in problem (6) as follows:

7 $$\begin{aligned} &t^{T}M_{0}t+\mathop{\max} _{\omega_{s}^{1}\in\Omega_{\infty}^{1}} \Biggl(\sum_{s = 1}^{S} {\omega_{s}^{1}t^{T}M_{s}t} \Biggr)+ \bigl(t^{T},y^{T} \bigr)\begin{pmatrix} M_{0}l+q_{0} \\ u-l \end{pmatrix} \\ & \quad{}+\mathop{\max} _{\omega_{s}^{1}\in\Omega_{\infty}^{1}} \Biggl( \sum_{s = 1}^{S} {\omega_{s}^{1}t^{T}M_{s}l} \Biggr) + \mathop{\max} _{\omega_{s}^{2}\in\Omega_{\infty}^{2}} \Biggl( \sum_{s = 1}^{S} {\omega_{s}^{2}t^{T}q_{s}} \Biggr)\leq z. \end{aligned}$$

Noting that $\Vert \eta \Vert _{1}=\mathop{\max} _{ \Vert \omega \Vert _{\infty}\leq1}\eta ^{T}\omega$, we evaluate the maximum in the above formulation as follows:

8 $$\begin{aligned} \mathop{\max} _{\omega_{s}^{1}\in\Omega_{\infty}^{1}} \Biggl(\sum _{s = 1}^{S} {\omega_{s}^{1}t^{T}M_{s}t} \Biggr) = \sum_{s = 1}^{S}\mathop {\max} _{\omega_{s}^{1}\in[0,1]} \bigl( \omega_{s}^{1}t^{T}M_{s}t \bigr) = \sum_{s = 1}^{S}\max \bigl(t^{T}M_{s}t,0 \bigr) = \sum _{s = 1}^{S}t^{T}M_{s}t, \end{aligned}$$

where the last equality is obtained by applying the positive semidefiniteness of $M_{s}$ for $s=1,\ldots,S$. Similarly, we have

9 $$\begin{aligned} & \mathop{\max} _{\omega_{s}^{1}\in\Omega_{\infty}^{1}} \Biggl(\sum _{s = 1}^{S} {\omega_{s}^{1}t^{T}M_{s}l} \Biggr)=\sum_{s = 1}^{S}t^{T}M_{s}l, \end{aligned}$$

10 $$\begin{aligned} & \mathop{\max} _{\omega_{s}^{2}\in\Omega_{\infty}^{2}} \Biggl(\sum _{s = 1}^{S} {\omega_{s}^{2}t^{T}q_{s}} \Biggr)= \left \Vert \begin{pmatrix} t^{T}q_{1} \\ \vdots\\ t^{T}q_{S} \end{pmatrix} \right \Vert _{1}=\sum _{s = 1}^{S} \bigl\vert t^{T}q_{s} \bigr\vert . \end{aligned}$$

Hence, reformulation (7) can be rewritten as:

$$\begin{aligned} t^{T} \Biggl[M_{0}+\sum_{s = 1}^{S}M_{s} \Biggr]t+ \bigl(t^{T},y^{T} \bigr)\begin{pmatrix} M_{0}l+q_{0} \\ u-l \end{pmatrix} +\sum _{s = 1}^{S}t^{T}M_{s}l+ \sum_{s = 1}^{S} \bigl\vert t^{T}q_{s} \bigr\vert \leq z. \end{aligned}$$

We then give the equivalent form of the second constraint in problem (6) as follows:

11 $$\begin{aligned} & \begin{bmatrix} M_{0} & I \\ -I & \mathbf{0} \end{bmatrix} _{j} \begin{pmatrix} t \\ y \end{pmatrix}+\mathop{\min} _{\omega_{s}^{1}\in\Omega_{\infty}^{1}} \begin{bmatrix} \sum_{s = 1}^{S} {\omega_{s}^{1} M_{s} } & \mathbf{0} \\ \mathbf{0} & \mathbf{0} \end{bmatrix} _{j}\begin{pmatrix} t \\ y \end{pmatrix} + \begin{pmatrix} M_{0}l+q_{0} \\ u-l \end{pmatrix} _{j} \\ &\quad{}+\mathop{\min} _{\omega_{s}^{1}\in\Omega _{\infty}^{1}}\begin{pmatrix} \sum_{s = 1}^{S} {\omega_{s}^{1} M_{s}l } \\ \mathbf{0} \end{pmatrix} _{j}+\mathop{ \min} _{\omega_{s}^{2}\in\Omega_{\infty}^{2}}\begin{pmatrix} \sum_{s = 1}^{S} {\omega_{s}^{2} q_{s} } \\ \mathbf{0} \end{pmatrix} _{j}\geq0,\quad j=1, \ldots,2n. \end{aligned}$$

After a simple calculation, for $i=1, \ldots, n, s=1, \ldots, S$, we have

$$\begin{aligned} &\quad\quad\mathop{\min} _{\omega_{s}^{1}\in\Omega_{\infty}^{1}} \begin{bmatrix} \sum_{s = 1}^{S} {\omega_{s}^{1} M_{s} } & \mathbf{0} \\ \mathbf{0} & \mathbf{0} \end{bmatrix} _{j} \begin{pmatrix} t \\ y \end{pmatrix} \\ &\quad =\sum_{s = 1}^{S}\mathop{\min} _{\omega_{s}^{1}\in[0,1]}\omega _{s}^{1} [M_{s} ]_{i}t=\sum_{s = 1}^{S}\min \bigl( [M_{s} ]_{i}t,0 \bigr)=-\sum_{s = 1}^{S} \max \bigl(- [M_{s} ]_{i}t,0 \bigr). \end{aligned}$$

Similarly, we have $\mathop{\min} _{\omega_{s}^{1}\in\Omega_{\infty }^{1}} (\sum_{s = 1}^{S} {\omega_{s}^{1} M_{s}l} )_{i}=-\sum_{s = 1}^{S}\max (- [M_{s} ]_{i}l,0 )$ and

12 $$\begin{aligned} \mathop{\min} _{\omega_{s}^{2}\in\Omega_{\infty}^{2}} \Biggl(\sum _{s = 1}^{S}\omega_{s}^{2} q_{s} \Biggr)_{i}=-\mathop{\max} _{\omega _{s}^{2}\in\Omega_{\infty}^{2}} \Biggl(- \sum_{s = 1}^{S}\omega_{s}^{2} q_{s} \Biggr)_{i}=-\sum_{s = 1}^{S} \bigl\vert ( q_{s} )_{i} \bigr\vert . \end{aligned}$$

By the fact that the upper bound $u\geq x$, we simplify formulation (11) as follows:

$$\begin{aligned} &[M_{0} ]_{i}t+y_{i}-{\sum _{s = 1}^{S}}\max \bigl(- [M_{s} ]_{i}t,0 \bigr)+ [M_{0} ]_{i}l+ (q_{0} )_{i} \\ &\quad{} -\sum_{s = 1}^{S}\max \bigl(- [M_{s} ]_{i}l,0 \bigr)-\sum_{s = 1}^{S} \bigl\vert (q_{s})_{i} \bigr\vert \geq0. \end{aligned}$$

Here, $i=1,\ldots,n$ and for a given vector $x\in R^{n}, \vert x \vert =( \vert x_{1} \vert ,\ldots, \vert x_{n} \vert )^{T}$. Then problem (6) can be rewritten as follows:

$$\begin{aligned} &\quad\min \quad\quad z \\ &\mbox{s.t.}\quad t^{T} \Biggl[M_{0}+\sum_{s = 1}^{S}M_{s} \Biggr]t+ \bigl(t^{T},y^{T} \bigr)\begin{pmatrix} M_{0}l+q_{0} \\ u-l \end{pmatrix} +\sum_{s = 1}^{S}t^{T}M_{s}l+ \sum_{s = 1}^{S} \bigl\vert t^{T}q_{s} \bigr\vert \leq z, \\ & \quad\quad[M_{0} ]_{i}t+y_{i}-{\sum _{s = 1}^{S}}\max \bigl(- [M_{s} ]_{i}t,0 \bigr)+ [M_{0} ]_{i}l+ (q_{0} )_{i} \\ &\quad\quad \qquad{}-\sum_{s = 1}^{S}\max \bigl(- [M_{s} ]_{i}l,0 \bigr)-\sum_{s = 1}^{S} \bigl\vert (q_{s})_{i} \bigr\vert \geq0,\quad i=1,\ldots,n, \\ & \quad\quad\quad t,y\geq0. \end{aligned}$$

Since it is difficult to compute maximum and absolute value functions, we can eliminate them by increasing relaxation variables $a_{s}\in R_{+}, \alpha_{s},\beta_{s}\in R_{+}^{n}, s=1,\ldots,S$. Then the above problem can be converted to QCQP:

$$\begin{aligned} &\min \quad\quad z \\ & \mbox{s.t.}\quad t^{T} \Biggl[M_{0}+\sum_{s = 1}^{S}M_{s} \Biggr]t+ \bigl(t^{T},y^{T} \bigr)\begin{pmatrix} M_{0}l+q_{0} \\ u-l \end{pmatrix} +\sum_{s = 1}^{S}t^{T}M_{s}l+ \sum_{s = 1}^{S}a_{s}\leq z, \\ &\quad\quad M_{0}t+M_{0}l+y+q_{0}-{\sum _{s = 1}^{S}}\alpha_{s}-{\sum _{s = 1}^{S}}\beta_{s}-\sum _{s = 1}^{S} \vert q_{s} \vert \geq0, \\ &\quad\quad{-}a_{s}\leq t^{T}q_{s}\leq a_{s}, \\ &\quad\quad M_{s}t+\alpha_{s}\geq0, \\ &\quad\quad M_{s}l+\beta_{s}\geq0, \\ &\quad\quad a_{s},\alpha_{s},\beta_{s}\geq0,\quad s=1,\ldots,S, \\ &\quad\quad t,y\geq0. \end{aligned}$$

Case 2: $\Omega^{2}=\Omega^{2}_{1}$.

We first replace $\omega_{s}^{2}\in\Omega_{\infty}^{2}$ in formulation (7) by $\omega_{s}^{2}\in\Omega_{1}^{2}$ and noting that $\Vert \eta \Vert _{\infty}=\mathop{\max} _{ \Vert \omega \Vert _{1}\leq1}\omega^{T}\eta$, then for $i=1,\ldots,n$, we have

13 $$\begin{aligned} \mathop{\max} _{\omega_{s}^{2}\in\Omega_{1}^{2}} \Biggl(\sum _{s = 1}^{S} {\omega_{s}^{2}t^{T}q_{s}} \Biggr) = \mathop{\max} _{\omega _{s}^{2}\in\Omega_{1}^{2}} \bigl(\omega^{2} \bigr)^{T}\begin{pmatrix} t^{T}q_{1} \\ \vdots\\ t^{T}q_{S} \end{pmatrix} = \left \Vert \begin{pmatrix} t^{T}q_{1} \\ \vdots\\ t^{T}q_{S} \end{pmatrix} \right \Vert _{\infty} = \mathop{\max} _{s=\{1,\ldots,S\}} \bigl\vert t^{T}q_{s} \bigr\vert \end{aligned}$$

and

14 $$\begin{aligned} \mathop{\min} _{\omega_{s}^{2}\in\Omega_{1}^{2}} \Biggl(\sum _{s = 1}^{S} {\omega_{s}^{2} q_{s} } \Biggr)_{i}=-\mathop{\max} _{\omega _{s}^{2}\in\Omega_{1}^{2}} \Biggl(- \sum_{s = 1}^{S} {\omega_{s}^{2} q_{s} } \Biggr)_{i}=-\mathop{\max} _{s\in\{1,\ldots,S\}} \bigl\vert (q_{s} )_{i} \bigr\vert . \end{aligned}$$

After a simple calculation, problem (6) can be rewritten as follows:

$$\begin{aligned} &\quad\mathop{\min} \quad\quad z \\ &\mbox{s.t.} \quad t^{T} \Biggl[M_{0} + \sum _{s = 1}^{S}M_{s} \Biggr] t + \bigl(t^{T},y^{T} \bigr)\begin{pmatrix} M_{0}l+q_{0} \\ u-l \end{pmatrix} + \sum _{s = 1}^{S}t^{T}M_{s}l + \mathop{ \max} _{s\in\{1,\ldots,S\}} \bigl\vert t^{T}q_{s} \bigr\vert \leq z, \\ &\quad\quad [M_{0}]_{i}t+y_{i}-\sum _{s = 1}^{S}\max \bigl(-[M_{s}]_{i}t,0 \bigr)+[M_{0}]_{i}l+[q_{0}]_{i} \\ & \quad\quad\quad{}-\sum_{s = 1}^{S}\max \bigl(-[M_{s}]_{i}l,0 \bigr)-\mathop{\max} _{s\in\{1,\ldots,S\}} \bigl\vert (q_{s})_{i} \bigr\vert \geq 0,\quad i=1,\ldots,n, \\ &\quad\quad\quad t,y\geq0. \end{aligned}$$

In order to calculate easily, we introduce variables $a\in R_{+},\alpha _{s},\beta_{s}\in R_{+}^{n},s=1,\ldots,S$. Then we obtain the following QCQP:

$$\begin{aligned} &\quad\min \quad\quad z \\ &\mbox{s.t.}\quad t^{T} \Biggl[M_{0}+\sum_{s = 1}^{S}M_{s} \Biggr]t+ \bigl(t^{T},y^{T} \bigr)\begin{pmatrix} M_{0}l+q_{0} \\ u-l \end{pmatrix} +\sum _{s = 1}^{S}t^{T}M_{s}l+a \leq z, \\ &\quad\quad M_{0}t+M_{0}l+y+q_{0}-\sum _{s = 1}^{S}\alpha_{s}-\sum _{s = 1}^{S}\beta_{s}- \vert q_{s} \vert \geq0, \\ &\quad\quad{-}a\leq t^{T}q_{s}\leq a, \\ &\quad\quad M_{s}t+\alpha_{s}\geq0, \\ &\quad\quad M_{s}l+\beta_{s}\geq0, \\ &\quad\quad \alpha_{s},\beta_{s}\geq0, \quad s=1,\ldots,S, \\ &\quad\quad\quad a,t,y\geq0. \end{aligned}$$

Case 3: $\Omega^{2}=\Omega_{2}^{2}$.

Firstly, we use $\omega_{s}^{2}\in\Omega_{2}^{2}$ instead of $\omega _{s}^{2}\in\Omega_{\infty}^{2}$ in formulation (7). By Example 1.3.3 in [19], it is seen that $\mathop{\max} _{ \Vert \omega \Vert _{2}\leq r}\omega^{T}\eta=r\frac{\eta^{T}\eta}{ \Vert \eta \Vert _{2}}=r \Vert \eta \Vert _{2}$, which implies that $\mathop{\max} _{ \Vert \omega \Vert _{2}\leq1}\omega ^{T}\eta= \Vert \eta \Vert _{2}$ and $\mathop{\min} _{ \Vert \omega \Vert _{2}\leq1}\omega ^{T}\eta=-\frac{\eta^{T}\eta}{ \Vert \eta \Vert _{2}}=- \Vert \eta \Vert _{2}$. As a result, for every $i=1,\ldots,n$, we have

15 $$\begin{aligned} \mathop{\max} _{\omega_{s}^{2}\in\Omega_{2}^{2}} \Biggl(\sum _{s = 1}^{S}\omega_{s}^{2}t^{T}q_{s} \Biggr)=\mathop{\max} _{\omega_{s}^{2}\in \Omega_{2}^{2}} \bigl(\omega^{2} \bigr)^{T}\begin{pmatrix} t^{T}q_{1} \\ \vdots\\ t^{T}q_{S} \end{pmatrix} = \left \Vert \begin{pmatrix} t^{T}q_{1} \\ \vdots\\ t^{T}q_{S} \end{pmatrix} \right \Vert _{2} =\sqrt{\sum _{s = 1}^{S} \bigl(t^{T}q_{s} \bigr)^{2}} \end{aligned}$$

and

16 $$\begin{aligned} \mathop{\min} _{\omega_{s}^{2}\in\Omega_{2}^{2}} \Biggl(\sum _{s = 1}^{S}\omega_{s}^{2} \bigl\vert (q_{s})_{i} \bigr\vert \Biggr) = \mathop{\min} _{\omega_{s}^{2}\in\Omega_{2}^{2}} \bigl(\omega^{2} \bigr)^{T} \begin{pmatrix} (q_{1})_{i} \\ \vdots\\ (q_{S})_{i} \end{pmatrix} = - \left \Vert \begin{pmatrix} (q_{1})_{i} \\ \vdots\\ (q_{S})_{i} \end{pmatrix} \right \Vert _{2} = -\sqrt{\sum_{s = 1}^{S} ( q_{s} )_{i}^{2}}. \end{aligned}$$

After a simple calculation, for $i=1,\ldots,n$, problem (6) can be rewritten as follows:

$$\begin{aligned} &\quad\min \quad\quad z \\ &\mbox{s.t.} \quad t^{T} \Biggl[M_{0}+\sum_{s = 1}^{S}M_{s} \Biggr] t + \bigl(t^{T},y^{T} \bigr) \begin{pmatrix} M_{0}+q_{0} \\ u-l \end{pmatrix} + \sum_{s = 1}^{S}t^{T}M_{s}l + \sqrt{\sum_{s = 1}^{S} \bigl(t^{T}q_{s} \bigr)^{2}}\leq z, \\ &\quad\quad[M_{0}]_{i}t+y_{i}-\sum _{s = 1}^{S}\max \bigl(-[M_{s}]_{i}t,0 \bigr)+[M_{0}]_{i}l+[q_{0}]_{i} \\ &\quad\quad\quad{} -\sum_{s = 1}^{S}\max \bigl(-[M_{s}]_{i}l,0 \bigr)-\sqrt{\sum _{s = 1}^{S} ( q_{s} )_{i}^{2}} \geq0, \\ &\quad\quad\quad t,y\geq0. \end{aligned}$$

Through adding variables $a\in R_{+},\alpha_{s},\beta_{s}\in R_{+}^{n},s=1,\ldots,S$, the above problem can be reformulated as the following convex program with a conic quadratic inequality quadratic program:

$$\begin{aligned} &\quad\min\quad\quad z \\ &\mbox{s.t.} \quad t^{T} \Biggl[M_{0}+\sum_{s = 1}^{S}M_{s} \Biggr]t+ \bigl(t^{T},y^{T} \bigr)\begin{pmatrix} M_{0}+q_{0} \\ u-l \end{pmatrix} +\sum _{s = 1}^{S}t^{T}M_{s}l+a \leq z, \\ &\quad\quad M_{0}t+M_{0}l+y+q_{0}-\sum _{s = 1}^{S}\alpha_{s}-\sum _{s = 1}^{S}\beta_{s}-\sqrt{\sum _{s = 1}^{S} ( q_{s} )^{2}}\geq0, \\ &\quad\quad\sqrt{\sum_{s = 1}^{S} \bigl(t^{T}q_{s} \bigr)^{2}}\leq a, \\ &\quad\quad M_{s}t+\alpha_{s}\geq0, \\ &\quad\quad M_{s}l+\beta_{s}\geq0, \\ &\quad\quad\alpha_{s},\beta_{s}\geq0,\quad s=1,\ldots S, \\ &\quad\quad a,t,y\geq0. \end{aligned}$$

These complete the proof. □

We now consider the robust counterpart of (6) defined by $\Omega ^{1}=\Omega^{1}_{1}$ and $\omega^{2}\in\Omega^{2}$.

### Theorem 2

*Consider the optimization problem* (6) *where* $\omega^{1}\in\Omega ^{1}_{1}$ *and* $M(\omega^{1})=M_{0}+\sum_{s = 1}^{S} {\omega_{s}^{1}M_{s} }, q(\omega^{2})=q_{0}+\sum_{s = 1}^{S} {\omega_{s}^{2} q_{s}}$. *Then* (6) *can be reformulated as QCQP or convex program with a conic quadratic inequality quadratic program while* $\omega^{2}$ *belongs to different sets of* $\Omega^{2}$.

### Proof

Case 1: $\Omega^{2}=\Omega_{\infty}^{2}$.

We first consider the equivalent form of quadratic constraint in problem (6), it can be represented as

17 $$\begin{aligned} &t^{T}M_{0}t+\mathop{\max} _{\omega_{s}^{1}\in\Omega_{1}^{1}} \Biggl(\sum_{s = 1}^{S}\omega_{s}^{1}t^{T}M_{s}t \Biggr)+ \bigl(t^{T},y^{T} \bigr)\begin{pmatrix} M_{0}l+q_{0} \\ u-l \end{pmatrix} \\ & \quad{}+\mathop{\max} _{\omega_{s}^{1}\in\Omega_{1}^{1}} \Biggl(\sum_{s = 1}^{S} \omega_{1}^{1}t^{T}M_{s}l \Biggr)+ \mathop{\max} _{\omega_{s}^{2}\in\Omega_{\infty}^{2}} \Biggl(\sum_{s = 1}^{S} \omega _{s}^{2}t^{T}q_{s} \Biggr)\leq z. \end{aligned}$$

We then have to evaluate the result of maximum in the above formulation by $\Vert \eta \Vert _{\infty}=\mathop{\max} _{ \Vert \omega \Vert _{1}\leq1}\eta ^{T}\omega$ that

18 $$\begin{aligned} \mathop{\max} _{\omega_{s}^{1}\in\Omega_{1}^{1}} \Biggl(\sum _{s = 1}^{S}\omega_{s}^{1}t^{T}M_{s}t \Biggr)=\mathop{\max} _{s\in\{1,\ldots ,S\}} \bigl(\mathop{\max} \bigl(t^{T}M_{s}t,0 \bigr) \bigr)=\mathop{\max} _{s\in\{1,\ldots,S\} }t^{T}M_{s}t. \end{aligned}$$

Here, we can obtain the last equality by applying the positive semidefiniteness of $M_{s}$ for $s=1,\ldots,S$. Similarly, we have

19 $$\begin{aligned} \mathop{\max} _{\omega_{s}^{1}\in\Omega_{1}^{1}} \Biggl(\sum _{s = 1}^{S}\omega_{s}^{1}t^{T}M_{s}l \Biggr)=\mathop{\max} _{s\in\{1,\ldots ,S\}}t^{T}M_{s}l. \end{aligned}$$

It then follows from (10) that formulation (17) can be rewritten as

$$\begin{aligned} t^{T}[M_{0}+M_{s}]t+ \bigl(t^{T},y^{T} \bigr)\begin{pmatrix} M_{0}l+q_{0} \\ u-l \end{pmatrix} +t^{T}M_{s}l+\sum _{s = 1}^{S} \bigl\vert t^{T}q_{s} \bigr\vert \leq z. \end{aligned}$$

We then give the equivalent component form of the second constraint in problem (6) by formulation (12) as follows:

$$\begin{aligned} &\!\!\!\!\!\!\!\!\!\!\!\! [ M_{0}]_{i}t + y_{i} + [M_{0}]_{i}l- \max_{s\in\{1,\ldots,S\}} \bigl(\max \bigl(-[M_{s}]_{i}t,0 \bigr) \bigr) - \max_{s\in\{1,\ldots,S\}} \bigl(\max \bigl(-[M_{s}]_{i}l,0 \bigr) \bigr)- \sum_{s = 1}^{S} \bigl\vert (q_{s})_{i} \bigr\vert \geq 0 \\ &\!\!\!\!\!\!\!\!\!\!\!\! \!\!\!\!\!\!\!\!\!\!\!\!\quad\Leftrightarrow\quad \max \bigl(-[M_{s}]_{i}t,0 \bigr)+\max \bigl(-[M_{s}]_{i}l,0 \bigr)\leq [M_{0}]_{i}t+y_{i}+[M_{0}]_{i}l+[q_{0}]_{i}- \sum_{s = 1}^{S} \bigl\vert (q_{s})_{i} \bigr\vert \\ &\!\!\!\!\!\!\!\!\!\!\!\! \!\!\!\!\!\!\!\!\!\!\!\! \quad\Leftrightarrow\quad \max(-M_{s}t,0)+\max(-M_{s}l,0)\leq M_{0}t+y+M_{0}l+q_{0}-\sum _{s = 1}^{S} \vert q_{s} \vert , \\ &\quad\quad\quad i=1,\ldots,n, s=1,\ldots,S. \end{aligned}$$

Finally, by adding variables $a_{s}\in R_{+},s=1,\ldots,S,\alpha,\beta \in R_{+}^{n}$, we obtain QCQP as follows:

$$\begin{aligned} &\quad\min\quad\quad z \\ &\mbox{s.t.}\quad t^{T}(M_{0}+M_{s})t+ \bigl(t^{T},y^{T} \bigr)\begin{pmatrix} M_{0}l+q_{0} \\ u-l \end{pmatrix} +t^{T}M_{s}l+\sum _{s = 1}^{S}a_{s}\leq z,\quad s=1,\ldots,S, \\ &\quad\quad M_{0}t+M_{0}l+y+q_{0}-\alpha-\beta-\sum _{s = 1}^{S} \vert q_{s} \vert \geq0, \\ &\quad\quad{-}a_{s}\leq t^{T}q_{s}\leq a_{s}, \\ &\quad\quad M_{s}t+\alpha\geq0, \\ &\quad\quad M_{s}l+\beta\geq0, \\ &\quad\quad a_{s}\geq0,\quad s=1,\ldots,S, \\ &\quad\quad \alpha,\beta,t,y \geq0. \end{aligned}$$

Inspired by Case 1, we derive the conclusions of Case 2 and Case 3.

Case 2: $\Omega^{2}=\Omega_{1}^{2}$.

We replace $\omega_{s}^{2}\in\Omega_{\infty}^{2}$ in formulation (17) by $\omega_{s}^{2}\in\Omega_{1}^{2}$ and combining with (13), (14), (18) and (19), by adding variables $a\in R_{+},\alpha,\beta\in R_{+}^{n}$, problem (6) can be reformulated as the following QCQP:

$$\begin{aligned} &\quad\min\quad\quad z \\ &\mbox{s.t.}\quad t^{T}(M_{0}+M_{s})t+ \bigl(t^{T},y^{T} \bigr)\begin{pmatrix} M_{0}l+q_{0} \\ u-l \end{pmatrix} +t^{T}M_{s}l+a\leq z, \\ &\quad\quad M_{0}t+M_{0}l+y+q_{0}-\alpha-\beta- \vert q_{s} \vert \geq0, \\ &\quad\quad {-}a\leq t^{T}q_{s}\leq a, \\ &\quad\quad M_{s}t+\alpha\geq0, \\ &\quad\quad M_{s}l+\beta\geq0,\quad s=1,\ldots,S, \\ &\quad\quad a,\alpha,\beta,t,y \geq0. \end{aligned}$$

Case 3: $\Omega^{2}=\Omega_{2}^{2}$.

We use $\omega_{s}^{2}\in\Omega_{2}^{2}$ instead of $\omega_{s}^{2}\in \Omega_{\infty}^{2}$ in formulation (17) and taking (15), (16), (18) and (19) into account, by adding variables $a\in R_{+},\alpha,\beta\in R_{+}^{n}$, problem (6) can be converted to convex program with a conic quadratic inequality quadratic program as follows:

$$\begin{aligned} &\quad\min \quad\quad z \\ &\mbox{s.t.}\quad t^{T}(M_{0}+M_{s})t+ \bigl(t^{T},y^{T} \bigr)\begin{pmatrix} M_{0}l+q_{0} \\ u-l \end{pmatrix} +t^{T}M_{s}l+a\leq z, \\ & \quad\quad M_{0}t+M_{0}l+y+q_{0}-\alpha-\beta-\sqrt {\sum_{s = 1}^{S} ( q_{s} )^{2}}\geq0, \\ &\quad\quad\sqrt{\sum_{s = 1}^{S} \bigl(t^{T}q_{s} \bigr)^{2}}\leq a, \\ &\quad\quad M_{s}t+\alpha\geq0, \\ &\quad\quad M_{s}l+\beta\geq0, \quad s=1,\ldots,S, \\ &\quad\quad a,\alpha,\beta,t,y \geq0. \end{aligned}$$

These complete the proof. □

Theorems 1 and 2 present an approach to seek robust solutions of $\operatorname{SLVI}(l,u,F)$, when $M_{s} (s=1,\ldots,S)$ is a positive semidefinite matrix. However, this condition is a little strict so that it is difficult to satisfy in practice. Thus, we give more general circumstances in Section 3.

## Tractable robust counterparts of non-monotone $\operatorname{SLVI}(l,u,F)$

In this section, we first define $M(\omega)=M_{0}+\sum_{s = 1}^{S} {\omega_{s}M_{s} }+\sum_{k = 1}^{K} {\omega_{S+k}M^{\prime}_{k} }$, where $M(\omega),M_{0},M_{s}$, $M_{k}^{\prime}$ are symmetric matrices for $s=1,\ldots,S, k=1,\ldots,K$; if not, we may replace the matrices by their symmetrical counterparts, and $M_{0},M_{s}\succeq0,s=1,\ldots ,S, M_{k}^{\prime}\preceq0,k=1,\ldots,K$. Then the corresponding function

$$\begin{aligned} \begin{bmatrix} M(\omega) & I \\ -I & \mathbf{0} \end{bmatrix} \begin{pmatrix} t \\ y \end{pmatrix} + \begin{pmatrix} M(\omega)l+q(\omega) \\ u-l \end{pmatrix} \end{aligned}$$

is no longer monotone for $\omega\in\Omega$, a.s. In this section, we consider the case that *q* is a certain vector. Since it is difficult to directly apply the results of quadratic program, these problems are somewhat more challenging. As we proceed, we may still obtain the results that robust problem (6) can be reformulated as QCQP or convex program under suitably defined uncertain sets.

### Theorem 3

*Consider the optimization problem* (6) *with uncertain sets defined by* $\Omega^{2}$, (6)* can be reformulated as QCQP or convex program*.

### Proof

Case 1: $\Omega=\Omega_{\infty}^{2}$.

Firstly, we consider the first constraint in formulation (6). Similar to (8), (9), combining with $\mathop{\max} _{ \Vert \omega \Vert _{\infty}\leq1}\eta^{T}\omega= \Vert \eta \Vert _{1}$ and $M_{s}\succeq 0, M_{k}^{\prime}\preceq0$, for every $s,k$, we have

$$\begin{aligned} &\mathop{\max} _{\omega\in\Omega_{\infty}^{2}} \Biggl(\sum_{s = 1}^{S} \omega_{s}t^{T}M_{s}t \Biggr)=\sum _{s = 1}^{S} \bigl\vert t^{T}M_{s}t \bigr\vert =\sum_{s = 1}^{S}t^{T}M_{s}t, \\ &\mathop{\max} _{\omega\in\Omega_{\infty}^{2}} \Biggl(\sum_{k = 1}^{K} \omega_{S+k}t^{T}M^{\prime}_{k}t \Biggr)=\sum _{k = 1}^{K} \bigl\vert t^{T}M^{\prime}_{k}t \bigr\vert =-\sum_{k = 1}^{K}t^{T}M^{\prime}_{k}t, \\ &\mathop{\max} _{\omega\in\Omega_{\infty}^{2}} \Biggl(\sum_{s = 1}^{S} \omega_{s}t^{T}M_{s}l \Biggr)=\sum _{s = 1}^{S} \bigl\vert t^{T}M_{s}l \bigr\vert =\sum_{s = 1}^{S}t^{T}M_{s}l, \\ &\mathop{\max} _{\omega\in\Omega_{\infty}^{2}} \Biggl(\sum_{k = 1}^{K} \omega_{S+k}t^{T}M^{\prime}_{k}l \Biggr)=\sum _{k = 1}^{K} \bigl\vert t^{T}M^{\prime}_{k}l \bigr\vert =-\sum_{k = 1}^{K}t^{T}M^{\prime}_{k}l. \end{aligned}$$

We then consider the second constraint in formulation (6). Since $\mathop{\max} _{ \Vert \omega \Vert _{\infty}\leq1}\eta^{T}\omega= \Vert \eta \Vert _{1}$, there holds

$$\begin{aligned} \mathop{\max} _{\omega\in\Omega_{\infty}^{2}} \Biggl(\sum_{s=1}^{S} \omega _{s} [-M_{s}t ] \Biggr)=\sum _{s=1}^{S} \vert {-}M_{s}t \vert =\sum _{s=1}^{S} \vert M_{s}t \vert . \end{aligned}$$

In the same way, we have

$$\begin{aligned} &\mathop{\max} _{\omega\in\Omega_{\infty}^{2}} \Biggl(\sum_{k=1}^{K} \omega_{S+k} \bigl[-M^{\prime}_{k}t \bigr] \Biggr)=\sum _{k=1}^{K} \bigl\vert {-}M^{\prime}_{k}t \bigr\vert =\sum_{k=1}^{K} \bigl\vert M^{\prime}_{k}t \bigr\vert , \\ &\mathop{\max} _{\omega\in\Omega_{\infty}^{2}} \Biggl(\sum_{s=1}^{S} \omega_{s} [-M_{s}l ] \Biggr)=\sum _{s=1}^{S} \vert {-}M_{s}l \vert =\sum _{s=1}^{S} \vert M_{s}l \vert , \\ &\mathop{\max} _{\omega\in\Omega_{\infty}^{2}} \Biggl(\sum_{k=1}^{K} \omega_{S+k} \bigl[-M^{\prime}_{k}l \bigr] \Biggr)=\sum _{k=1}^{K} \bigl\vert {-}M^{\prime}_{k}l \bigr\vert =\sum_{k=1}^{K} \bigl\vert M^{\prime}_{k}l \bigr\vert . \end{aligned}$$

It then follows from the fact $h(x,\omega)\geq0, \omega\in\Omega_{\infty }^{2}$, a.s. $\Leftrightarrow\mathop{\min} _{\omega\in\Omega_{\infty }^{2}} h(x,\omega)\geq0$ and eliminate all absolute value by adding extra variables $\alpha_{s},\beta_{s}\in R_{+}^{n},s=1,\ldots,S+K$, then problem (6) can be rewritten as the following QCQP:

$$\begin{aligned} &\quad\min\quad\quad z \\ &\mbox{s.t.} \quad t^{T} \Biggl[ M_{0} + \sum _{s=1}^{S}M_{s} - \sum _{k=1}^{K}M^{\prime}_{k} \Biggr] t + \bigl(t^{T},y^{T} \bigr)\begin{pmatrix} M_{0}l+q \\ u-l \end{pmatrix} \\ &\quad\quad\qquad{} + t^{T} \Biggl[\sum_{s=1}^{S}M_{s} - \sum_{s=k}^{K}M^{\prime }_{k} \Biggr]l\leq z, \\ &\quad\quad M_{0}t+y-\sum_{s=1}^{S+K} \alpha_{s}+M_{0}l+q-\sum_{s=1}^{S+K} \beta _{s}\geq0, \\ &\quad\quad{-}\alpha_{s}\leq M_{s}t\leq\alpha_{s}, \\ &\quad\quad{-}\beta_{s}\leq M_{s}l\leq\beta_{s},\quad s=1, \ldots,S, \\ &\quad\quad{-}\alpha_{s}\leq M^{\prime}_{s-S}t\leq \alpha_{s}, \\ &\quad\quad{-}\beta_{s}\leq M^{\prime}_{s-S}l\leq \beta_{s},\quad s=S+1,\ldots,S+K, \\ &\quad\quad\alpha_{s},\beta_{s}\geq0,\quad s=1,\ldots,S+K, \\ &\quad\quad t,y\geq0. \end{aligned}$$

Case 2: $\Omega=\Omega_{1}^{2}$.

Similar to obtaining (18), (19), taking $\mathop{\max} _{ \Vert \omega \Vert _{1}\leq1}\omega^{T}\eta= \Vert \eta \Vert _{\infty}$ together with the fact that for all $t, l, t^{T}M_{s}t, t^{T}M_{s}l\geq0, s=1,\ldots ,S$ and $t^{T}M_{k}^{\prime}t, t^{T}M_{k}^{\prime}l\leq0, k=1,\ldots ,K$, we have

$$\begin{aligned} &\mathop{\max} _{\omega\in\Omega_{1}^{2}} \Biggl(\sum_{s = 1}^{S} \omega _{s}t^{T}M_{s}t+\sum _{k = 1}^{K}\omega_{S+k}t^{T}M^{\prime}_{k}t \Biggr)=\max \Bigl(\mathop{\max} _{s\in\{1,\ldots,S\}} \bigl\vert t^{T}M_{s}t \bigr\vert ,\mathop{\max} _{k\in\{1,\ldots,K\}} \bigl\vert t^{T}M^{\prime}_{k}t \bigr\vert \Bigr), \\ &\mathop{\max} _{\omega\in\Omega_{1}^{2}} \Biggl(\sum_{s = 1}^{S} \omega _{s}t^{T}M_{s}l+\sum _{k = 1}^{K}\omega_{S+k}t^{T}M^{\prime}_{k}l \Biggr)=\max \Bigl(\mathop{\max} _{s\in\{1,\ldots,S\}} \bigl\vert t^{T}M_{s}l \bigr\vert ,\mathop{\max} _{k\in\{1,\ldots,K\}} \bigl\vert t^{T}M^{\prime}_{k}l \bigr\vert \Bigr). \end{aligned}$$

Then the first constraint in problem (6) can be transformed as follows:

$$\begin{aligned} &t^{T}M_{0}t+\mathop{\max} _{s\in\{1,\ldots,S\}}t^{T}M_{s}t+ \bigl(t^{T},y^{T} \bigr)\begin{pmatrix} M_{0}l+q \\ u-l \end{pmatrix} +\mathop{\max} _{s\in\{1,\ldots,S\}}t^{T}M_{s}l\leq z, \\ &t^{T}M_{0}t+\mathop{\max} _{k\in\{1,\ldots,K\}} \bigl(-t^{T}M^{\prime }_{k}t \bigr)+ \bigl(t^{T},y^{T} \bigr)\begin{pmatrix} M_{0}l+q \\ u-l \end{pmatrix} + \mathop{\max} _{s\in\{1,\ldots,S\}}t^{T}M_{s}l\leq z, \\ &t^{T}M_{0}t+\mathop{\max} _{s\in\{1,\ldots,S\}}t^{T}M_{s}t+ \bigl(t^{T},y^{T} \bigr)\begin{pmatrix} M_{0}l+q \\ u-l \end{pmatrix} + \mathop{\max} _{k\in\{1,\ldots,K\}} \bigl(-t^{T}M^{\prime }_{k}l \bigr)\leq z, \\ &t^{T}M_{0}t+ \mathop{\max} _{k\in\{1,\ldots,K\}} \bigl(-t^{T}M^{\prime }_{k}t \bigr)+ \bigl(t^{T},y^{T} \bigr)\begin{pmatrix} M_{0}l+q \\ u-l \end{pmatrix} + \mathop{\max} _{k\in\{1,\ldots,K\}} \bigl(-t^{T}M^{\prime }_{k}l \bigr)\leq z. \end{aligned}$$

On the other hand, it follows from $\mathop{\max} _{ \Vert \omega \Vert _{1}\leq 1}\omega^{T}\eta= \Vert \eta \Vert _{\infty}$ that, for every $s,k$, we can deduce that

20 $$\begin{aligned} &\quad \mathop{\max} _{\omega\in\Omega_{1}^{2}} \Biggl(\sum_{s=1}^{S} \omega _{s} (-M_{s}t )+ \sum_{k=1}^{K} \omega_{S+k} \bigl(-M^{\prime}_{k}t \bigr) \Biggr) \\ &=\mathop{\max} _{\omega\in\Omega_{1}^{2}}\omega^{T} \begin{pmatrix} -M_{1}t \\ \vdots\\ -M_{S}t \\ -M_{1}^{\prime}t \\ \vdots\\ -M_{K}^{\prime}t \end{pmatrix} =\max \Bigl(\mathop{\max} _{s\in\{1,\ldots,S\}} \bigl( \vert {-}M_{s}t \vert \bigr),\mathop{\max} _{k\in\{1,\ldots,K\}} \bigl( \bigl\vert {-}M^{\prime }_{k}t \bigr\vert \bigr) \Bigr). \end{aligned}$$

Similarly, we have

21 $$\begin{aligned} &\mathop{\max} _{\omega\in\Omega_{1}^{2}} \Biggl(\sum _{s=1}^{S}\omega _{s} (-M_{s}l )+ \sum_{k=1}^{K}\omega_{S+k} \bigl(-M^{\prime}_{k}l \bigr) \Biggr) =\max \Bigl( \mathop{\max} _{s\in\{1,\ldots,S\}} \bigl( \vert {-}M_{s}l \vert \bigr), \mathop{\max} _{k\in\{1,\ldots,K\}} \bigl( \bigl\vert {-}M^{\prime}_{k}l \bigr\vert \bigr) \Bigr). \end{aligned}$$

It then follows from (20), (21) that the second constraint can be transformed as follows:

$$\begin{aligned} &M_{0}t+y-\mathop{\max} _{s\in\{1,\ldots,S\}} \bigl( \vert {-}M_{s}t \vert \bigr)+M_{0}l+q-\mathop{\max} _{s\in\{1,\ldots,S\}} \bigl( \vert {-}M_{s}l \vert \bigr)\geq0, \\ &M_{0}t+y-\mathop{\max} _{k\in\{1,\ldots,K\}} \bigl( \bigl\vert {-}M^{\prime }_{k}t \bigr\vert \bigr)+M_{0}l+q- \mathop{\max} _{s\in\{1,\ldots,S\}} \bigl( \vert {-}M_{s}l \vert \bigr) \geq0, \\ &M_{0}t+y-\mathop{\max} _{s\in\{1,\ldots,S\}} \bigl( \vert {-}M_{s}t \vert \bigr)+M_{0}l+q-\mathop{\max} _{k\in\{1,\ldots,K\}} \bigl( \bigl\vert {-}M^{\prime }_{k}l \bigr\vert \bigr)\geq0, \\ &M_{0}t+y-\mathop{\max} _{k\in\{1,\ldots,K\}} \bigl( \bigl\vert {-}M^{\prime }_{k}t \bigr\vert \bigr)+M_{0}l+q- \mathop{\max} _{k\in\{1,\ldots,K\}} \bigl( \bigl\vert {-}M^{\prime}_{k}l \bigr\vert \bigr)\geq0. \end{aligned}$$

Finally, in order to eliminate the maximum function, we add extra variables $\alpha_{1},\alpha_{2},\beta_{1}, \beta_{2}\in R_{+}^{n}$ into constraints. In addition, taking the fact $h(x,\omega)\geq0, \omega\in \Omega_{1}^{2}, \mbox{ a.s. }\Leftrightarrow \mathop{\min} _{\omega\in\Omega _{1}^{2}} h(x,\omega)\geq0$ into account, we obtain the QCQP form of problem (6) as follows:

$$\begin{aligned} &\quad \min \quad \quad z \\ &\mbox{s.t.}\quad t^{T}[M_{0}+M_{s}]t+ \bigl(t^{T},y^{T} \bigr)\begin{pmatrix} [M_{0}+M_{s}]l+q \\ u-l \end{pmatrix} \leq z, \\ &\quad \quad t^{T} \bigl[M_{0}-M^{\prime}_{k} \bigr]t+ \bigl(t^{T},y^{T} \bigr)\begin{pmatrix} [M_{0}+M_{s}]l+q \\ u-l \end{pmatrix} \leq z, \\ &\quad \quad t^{T}[M_{0}+M_{s}]t+ \bigl(t^{T},y^{T} \bigr)\begin{pmatrix} [M_{0}-M^{\prime}_{k}]l+q \\ u-l \end{pmatrix} \leq z, \\ &\quad \quad t^{T} \bigl[M_{0}-M^{\prime}_{k} \bigr]t+ \bigl(t^{T},y^{T} \bigr)\begin{pmatrix} [M_{0}-M^{\prime}_{k}]l+q \\ u-l \end{pmatrix} \leq z, \\ &\quad \quad M_{0}t-\alpha_{1}+M_{0}l+q- \beta_{1}\geq0, \\ &\quad \quad M_{0}t-\alpha_{1}+M_{0}l+q- \beta_{2}\geq0, \\ &\quad \quad M_{0}t-\alpha_{2}+M_{0}l+q- \beta_{1}\geq0, \\ &\quad \quad M_{0}t-\alpha_{2}+M_{0}l+q- \beta_{2}\geq0, \\ &\quad \quad {-}\alpha_{1}\leq M_{s}t\leq\alpha_{1}, \\ &\quad \quad {-}\beta_{1}\leq M_{s}l\leq\beta_{1},\quad s=1, \ldots,S, \\ &\quad \quad {-}\alpha_{2}\leq M^{\prime}_{k}t\leq \alpha_{2}, \\ &\quad \quad {-}\beta_{2}\leq M^{\prime}_{k}l\leq \beta_{2},\quad k=1,\ldots,K, \\ &\quad \quad \alpha_{1},\alpha_{2},\beta_{1}, \beta_{2},t,y\geq0. \end{aligned}$$

Case 3: $\Omega=\Omega_{2}^{2}$.

In this circumstance, we begin by simplifying the first constraint in problem (6), according to $\mathop{\max} _{ \Vert \omega \Vert _{2}\leq 1}\omega^{T}\eta= \Vert \eta \Vert _{2}$, we can derive that

$$\begin{aligned} &\quad \mathop{\max} _{\omega\in\Omega_{2}^{2}} \Biggl(\sum_{s = 1}^{S} \omega _{s}t^{T}M_{s}t+\sum _{k = 1}^{K}\omega_{S+k}t^{T}M^{\prime}_{k}t \Biggr) \\ &=\mathop{\max} _{\omega\in\Omega_{2}^{2}}\omega^{T}\begin{pmatrix} t^{T}M_{1}t \\ \vdots\\ t^{T}M_{S}t\\ t^{T}M^{\prime}_{1}t\\ \vdots\\ t^{T}M^{\prime}_{K}t \end{pmatrix}=\sqrt {\sum_{s = 1}^{S} \bigl(t^{T}M_{s}t \bigr)^{2}+\sum _{k = 1}^{K} \bigl(t^{T}M^{\prime}_{k}t \bigr)^{2}}. \end{aligned}$$

Similarly, we have

$$\begin{aligned} \mathop{\max} _{\omega\in\Omega_{2}^{2}} \Biggl(\sum_{s = 1}^{S} \omega _{s}t^{T}M_{s}l+\sum _{k = 1}^{K}\omega_{S+k}t^{T}M^{\prime}_{k}l \Biggr)=\sqrt{\sum_{s = 1}^{S} \bigl(t^{T}M_{s}l \bigr)^{2}+\sum _{k = 1}^{K} \bigl(t^{T}M^{\prime}_{k}l \bigr)^{2}}. \end{aligned}$$

Noting that, by calculating the second derivative of $\sqrt{\sum_{s = 1}^{S}(t^{T}M_{s}t)^{2}+\sum_{k = 1}^{K}(t^{T}M^{\prime}_{k}t)^{2}}$ and $\sqrt{\sum_{s = 1}^{S}(t^{T}M_{s}l)^{2}+\sum_{k = 1}^{K}(t^{T}M^{\prime}_{k}l)^{2}}$, we can easily derive that the first constraint is a convex constraint.

We then give the equivalent form of the second constraint in problem (6), according to $\mathop{\max} _{ \Vert \omega \Vert _{2}\leq1}\omega ^{T}\eta= \Vert \eta \Vert _{2}$, the formulation is transformed as follows:

$$\begin{aligned} &\quad \mathop{\max} _{\omega\in\Omega_{2}^{2}} \Biggl(\sum_{s = 1}^{S} \omega _{s}[M_{s}]_{i}t+\sum _{k = 1}^{K}\omega_{S+k} \bigl[M^{\prime }_{k} \bigr]_{i}t \Biggr) \\ & =\mathop{\max} _{\omega\in\Omega_{2}^{2}}\omega^{T}\begin{pmatrix} (M_{1})_{i}t \\ \vdots\\ (M_{S})_{i}t \\ (M^{\prime}_{1})_{i}t \\ \vdots\\ (M^{\prime}_{K})_{i}t \end{pmatrix} =\sqrt {\sum_{s = 1}^{S} \bigl([M_{s}]_{i}t \bigr)^{2}+\sum _{k = 1}^{K} \bigl( \bigl[M^{\prime }_{k} \bigr]_{i}t \bigr)^{2}},\quad i=1,\ldots,n. \end{aligned}$$

Noting that above *n* constraints are in the form of second-order cone constraints, they are tractable convex constraints. Similarly, for $i=1,\ldots,n$, we have

$$\begin{aligned} \mathop{\max} _{\omega\in\Omega_{2}^{2}} \Biggl(\sum_{s = 1}^{S} \omega _{s}[M_{s}]_{i}l+\sum _{k = 1}^{K}\omega_{S+k} \bigl[M^{\prime }_{k} \bigr]_{i}l \Biggr) =\sqrt{\sum _{s = 1}^{S} \bigl([M_{s}]_{i}l \bigr)^{2}+\sum_{k = 1}^{K} \bigl( \bigl[M^{\prime }_{k} \bigr]_{i}l \bigr)^{2}}. \end{aligned}$$

Therefore, problem (6) can be converted to a convex program as follows:

$$\begin{aligned} &\quad \min\quad \quad z \\ & \mbox{s.t.}\quad t^{T}M_{0}t+\sqrt{\sum _{s = 1}^{S} \bigl(t^{T}M_{s}t \bigr)^{2}+\sum_{k = 1}^{K} \bigl(t^{T}M^{\prime}_{k}t \bigr)^{2}}+ \bigl(t^{T},y^{T} \bigr)\begin{pmatrix} M_{0}l+q \\ u-l \end{pmatrix} \\ &\phantom{\quad \mbox{s.t.}\quad}\quad \quad \quad{}+\sqrt{\sum_{s = 1}^{S} \bigl(t^{T}M_{s}l \bigr)^{2}+\sum _{k = 1}^{K} \bigl(t^{T}M^{\prime}_{k}l \bigr)^{2}}\leq z, \\ &\quad \quad [M_{0}]_{i}t+y_{i}-\sqrt{ \sum_{s = 1}^{S} \bigl([M_{s}]_{i}t \bigr)^{2}+\sum_{k = 1}^{K} \bigl( \bigl[M^{\prime}_{k} \bigr]_{i}t \bigr)^{2}}+[M_{0}]_{i}l+q_{i} \\ &\quad \quad \quad \quad \quad{}-\sqrt{\sum_{s = 1}^{S} \bigl([M_{s}]_{i}l \bigr)^{2}+\sum _{k = 1}^{K} \bigl( \bigl[M^{\prime }_{k} \bigr]_{i}l \bigr)^{2}}\geq0, \quad i=1,\ldots,n, \\ &\quad \quad \quad t,y\geq0. \end{aligned}$$

The proof is completed. □

The core idea of Theorem 3 is to derive the tractable robust counterparts of $\operatorname{SLVI}(l,u,F)$. The case with uncertain *q* can be easily proved similar to Section 2, so it is omitted here.

## Conclusions

We present the first attempt to give the robust reformulation for solving the box-constrained stochastic linear variational inequality problem. For three types of uncertain variables, the robust reformulation of $\operatorname{SLVI}(l,u,F)$ can be solved as either a quadratically constrained quadratic program (QCQP) or a convex program, which are all more tractable and can provide solutions for $\operatorname{SLVI}(l,u,F)$, no matter for monotone or non-monotone *F*.
